# Genomic selection validated across two generations of loblolly pine breeding

**DOI:** 10.1093/g3journal/jkag122

**Published:** 2026-05-11

**Authors:** Fikret Isik, Mohammad Nasir Shalizi, Trevor D Walker

**Affiliations:** Department of Forestry and Environmental Resources, Cooperative Tree Improvement Program, North Carolina State University, Raleigh, NC 27695, United States; Department of Forestry and Environmental Resources, Cooperative Tree Improvement Program, North Carolina State University, Raleigh, NC 27695, United States; Department of Forestry and Environmental Resources, Cooperative Tree Improvement Program, North Carolina State University, Raleigh, NC 27695, United States

**Keywords:** tree breeding, genomic prediction, GenPed, shared data resource, *Pinus taeda*, ABLUP, ssGBLUP, prediction accuracy, relatedness, genetic gain

## Abstract

This study evaluated the effectiveness of genomic selection (GS) in loblolly pine (*Pinus taeda*) using a 2-generation closed breeding population and a large, genetically diverse Mainline population. Single-step genomic best linear unbiased prediction (ssGBLUP) models were used to include all phenotypic, genotypic, and pedigree information. Prediction accuracies of genomic estimated breeding values reached up to 0.70 for stem volume and stem straightness. Prediction accuracy showed a strong linear relationship with mean relatedness between training and validation populations (*r* > 0.92). Scaling the genomic and pedigree relationship matrices improved model stability, increased prediction accuracy, and reduced bias in genomic estimated breeding values. Estimates of heritability and variance components from ssGBLUP were consistent with pedigree-based models, particularly when genomic relationships were properly scaled. Genomic selection had approximately 50% more genetic gain per year relative to conventional selection. Overall, these results demonstrate that GS can be effectively integrated into operational *Pinus taeda* breeding programs, given sustained investment in large, well-connected training populations with high-quality phenotypic data. We also outline the planned implementation of GS in the North Carolina State University Cooperative Tree Improvement Program to increase genetic gain.

## Introduction

Genomic selection (GS) refers to predicting breeding values for individuals without phenotypic records using genome-wide DNA markers. Unlike early marker–trait association approaches that focused on a small number of loci with large effects, GS embraces the polygenic architecture of quantitative traits by modeling the covariance among relatives rather than individual marker effects. Since its introduction (Meuwissen et al. 2001), GS has triggered a paradigm shift in plant and animal breeding. In US dairy cattle, for example, widespread adoption of GS has doubled the rate of genetic gain since 2010 (Wiggans and Carrillo 2022). GS has also been successfully integrated into major crop improvement programs, including maize, wheat, and rice (Dreisigacker et al. 2021; da Silva et al. 2025).

For perennial, outcrossing woody species like forest trees, long generation intervals substantially delay genetic improvement when relying on conventional progeny testing. By reducing the need for progeny testing, GS can accelerate breeding cycles and increase gain per unit time. Early reviews highlighted the promise of GS for forest trees (Grattapaglia 2014; Isik 2014), and numerous proof-of-concept studies using intra-generational cross-validation methods demonstrated encouraging results (eg Resende et al. 2012; Ratcliffe et al. 2015; Durán et al. 2017; Lauer et al. 2022; Shalizi et al. 2022). These early studies partitioned a single generation into training and prediction sets to mimic the application of GS, which can overestimate prediction accuracy because marker–QTL linkage phases are largely preserved between subsets (Grattapaglia 2022; Isik 2022).

A more realistic assessment of GS performance uses earlier generations as the training population and their descendants as the validation population (Bartholomé et al. 2016). This approach accounts for the effects of recombination, which disrupts marker–QTL associations across generations, and therefore better reflects operational GS performance in true breeding programs. Despite growing interest in GS for forest trees, true across-generation validation remains rare, with only a few recent examples (Simiqueli et al. 2023; Duarte et al. 2024). Reported accuracies vary widely and are influenced by training population size, phenotypic data quality, and the degree of genetic relatedness between training and validation sets. Among these factors, relatedness plays a critical role in GS prediction accuracy but has not been thoroughly quantified in operational breeding contexts (Isik 2022). Clear guidelines are therefore needed to define appropriate training populations and to understand the limitations of GS when applied across generations in species with long breeding cycles.

Historically, GS was implemented using multi-stage genetic evaluation pipelines (VanRaden et al. 2009). In these approaches, pedigree-based best linear unbiased prediction (ABLUP) was first used to estimate breeding values, which were then converted into pseudo-phenotypes for genomic prediction (GBLUP). Genomic breeding values for genotyped individuals were subsequently combined with pedigree-based estimates using selection indices (Christensen et al. 2012; Bermann et al. 2022). These multi-stage approaches can introduce bias, reduce prediction accuracy, and do not fully utilize information for related but non-genotyped individuals (Misztal et al. 2009).

To address these limitations, single-step genomic best linear unbiased prediction (ssGBLUP) was proposed (Legarra et al. 2009; Christensen and Lund 2010), which integrates phenotypic, pedigree, and genotypic information into a unified framework (Lourenco et al. 2020). Compared with conventional ABLUP or 2-stage GBLUP approaches, ssGBLUP is simpler to implement, reduces bias between genotyped and non-genotyped individuals, accounts for selection on Mendelian sampling within families, and improves prediction accuracy (Misztal and Legarra 2017; Lourenco et al. 2020; Mäntysaari et al. 2020). Advances in computational efficiency, including sparse matrix algorithms and faster construction of the inverse hybrid relationship matrix, have enabled routine application of ssGBLUP to large-scale breeding datasets, making it a standard method in modern plant and animal breeding programs (Misztal et al. 2021; Bermann et al. 2022). The value of ssGBLUP has been demonstrated in forest tree breeding (Ukrainetz and Mansfield 2020; Walker et al. 2022; Gonzalez et al. 2025), but its implementation in operational tree breeding programs has yet to be realized.

For ssGBLUP to perform optimally, genomic relationships derived from SNP markers must be compatible with pedigree-based relationships for genotyped individuals (Legarra et al. 2014). Incompatibility arises primarily because allele frequencies of the base population, a theoretical group of unrelated individuals that serve as founders, are unknown and are typically approximated using the genotyped population's allele frequencies (Vitezica et al. 2011; Christensen et al. 2012). This can lead to biased estimates of variance components and breeding values (Forni et al. 2011). Several approaches have been proposed to make the relationships compatible, including regressing genomic relationships toward pedigree relationships (VanRaden 2008), adjusting the genomic relationship matrix by adding the average difference between pedigree and genomic relationships (Vitezica et al. 2011), and introducing meta-founders to explicitly model base population relationships, analogous to genetic groups in animal models (Westell et al. 1988; Christensen et al. 2012; Legarra et al. 2015). The evolution of these methods and their implications for ssGBLUP has been comprehensively reviewed by (Bermann et al. 2022). However, their application in tree breeding remains limited.

In this study, we evaluated GS using a multi-generational population of loblolly pine (*Pinus taeda*) developed by the Cooperative Tree Improvement Program at North Carolina State University. This closed breeding population, founded with 21 parents, was bred over 2 generations (2001 to 2025), and has pedigree connections with a larger, more diverse Mainline population. The objectives of the study were to:

1. Validate GS in a 2-generational breeding population by comparing its efficiency with conventional selection in terms of genetic gain per unit time.
2. Assess how relatedness between training and validation sets and training set size influence prediction accuracy using a large operational breeding population; and
3. Evaluate the effect of different weights assigned to pedigree- and marker-based relationships in ssGBLUP to address bias of genomic estimated breeding values (GEBVs).

## Materials and methods

### Plant material

#### First-generation Atlantic Coastal Elite population

The first-generation Atlantic Coastal Elite (ACE1) population was developed from 3 disconnected 8-parent diallels (Shalizi and Isik 2019). In total, 24 founding parents were mated to produce 76 full-sib families. Seedlings were artificially inoculated in 2007 with a mixed inoculum of *Cronartium quercuum* f. sp. *fusiforme*, the causal agent of fusiform rust disease. Individuals that developed stem galls were culled, and the remaining 2,488 gall-free seedlings, representing 51 full-sib families from 21 parents and 7 pollen-mix families were clonally propagated via rooted cuttings.

Eight field trials were established in 2009 and 2010 using an incomplete row–column design. Each clone was represented by a single copy (ramet) per site. Additionally, 443 seedlings from a checklot family were included across trials, bringing the total number of planted trees to 14,496 (Table 1). The ACE1 field trials were assessed in 2016, 6 yr after establishment.

**Table 1.** Summary of population structure for the Atlantic Coastal Elite (ACE1 and ACE2) and Coastal Mainline populations of loblolly pine (*Pinus taeda*), including the number of field tests, total number of trees, parents, full-sib crosses, and the distribution of genotyped and non-genotyped individuals used for genetic evaluation.

	ACE1	ACE2	Mainline
Field tests	8	4	60
Trees	14,496	3639	47,991
Parents	28	85	497
Crosses	51	73	813
Genotyped trees	2076	2108	6140
Non-genotyped trees	855	1531	41,851

#### Second-generation Atlantic Coastal Elite population (ACE2)

In 2017, 73 ACE1 clones were selected with emphasis on growth rate and stem form and mated to produce 67 full-sibling families for progeny testing (second-generation Atlantic Coastal Elite population [ACE2]). To enhance genetic connectivity with the Mainline population, the ACE2 field trials also included 6 full-sib crosses among ACE1 clones and parents from the Mainline population, as well as the same 7 pollen-mix families used in the previous ACE1 generation. Approximately 3,639 seedling progeny from these families were planted at 4 locations in 2021 using incomplete row–column designs (Table 1, Supplementary Fig. 1).

#### Mainline coastal breeding population

**Fig. 1.**
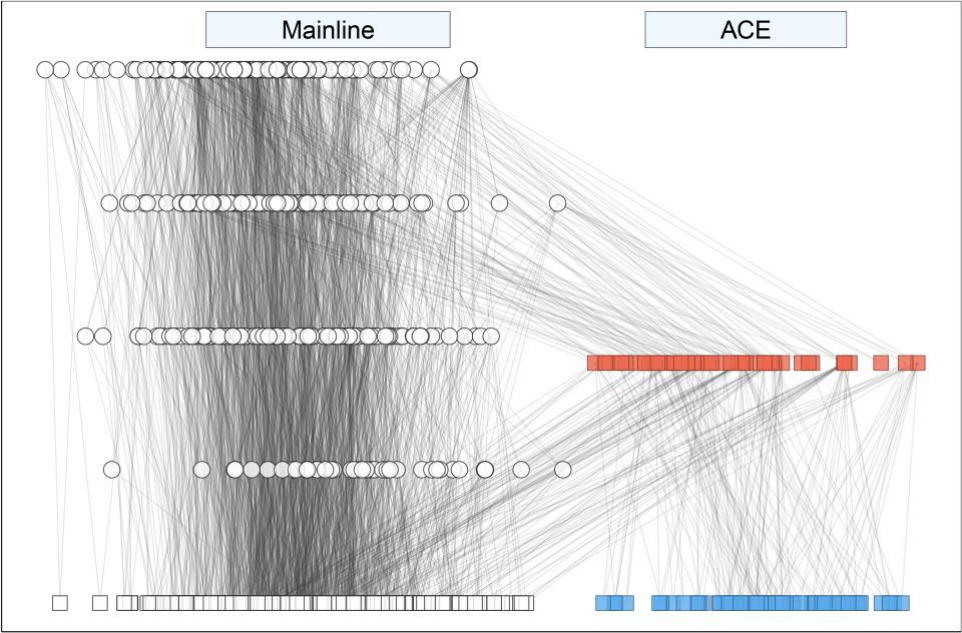
Pedigree diagram illustrating genetic relationships and diversity within and between the Mainline and ACE populations; circles represent parents and squares represent crosses.

The Atlantic Coastal Mainline fourth-cycle breeding population consists of about 497 parents. These parents were mated between 2011 and 2019 to produce 813 full-sib families (Isik and McKeand 2019). Progeny tests were established at 60 sites between 2014 and 2022, each containing about 120 families replicated in 6 to 20 blocks using incomplete row–column designs (Table 1). On average, a full-sib family had around 60 progeny at planting. The Mainline population shares pedigree connections with the ACE populations described before, including 28 and 40 parents shared with ACE1 and ACE2 populations, respectively, as well as common ancestors (Fig. 1).

#### Phenotyping

The ACE1 trials were assessed at age 6 yr and the ACE2 and Mainline population trials were assessed at age 4 yr. Measurements included individual-tree height (m), diameter at breast height (cm), stem straightness (1 to 6, with 1 indicating the straightest stems), incidence of fusiform rust disease (yes or no), and occurrence of stem forking (yes or no). Outside bark stem volume (dm^3^) was calculated using the volume equations (Sherrill et al. 2008). Approximately, 14,496 trees were evaluated in the ACE1 trials, 3,639 in the ACE2 trials, and 47,991 in the Mainline trials. In this study, stem volume and stem straightness were analyzed as the response variables.

#### Genotyping

In total, 10,324 trees from the ACE1, ACE2, and Mainline populations were genotyped, along with 213 founder and ancestral trees (Table 1). Diploid needle tissue samples were collected from the fresh growth of trees. Needle samples were placed in labeled coin envelopes and stored with silica desiccant to preserve freshness. Once dried, approximately 10 mg of tissue was prepared for DNA extraction. Trees were genotyped using the Pita50K Axiom SNP array developed for loblolly pine (Caballero et al. 2021). SNP markers were filtered based on a minor allele frequency threshold of 0.01 and a maximum missing rate of 10%. Samples with a no-call rate exceeding than 10% were excluded from further analysis. After filtering, approximately 15,000 SNP markers were retained with 9.2% of genotypes call missing. Missing genotypes were imputed using the “Wright” method of the *snpReady* package (Granato et al. 2018) in R statistical environment (R Core Team 2024).

#### Statistical models

Pedigree-based linear mixed models (ABLUP) were fitted to estimate variance components and obtain estimated breeding values (EBVs). Single-step genomic best linear unbiased prediction (ssGBLUP) models were fitted using the same fixed and random effects to estimate variance components and genomic estimated breeding values (GEBVs).

#### Predictions of EBVs using ABLUP

The following individual-tree (animal) model was fitted to predict EBVs on a common scale using data from 2 generations of the ACE population and the fourth-cycle Mainline breeding population:


(1) y=Xb+Zu+e

where y is the vector of observations; X and Z are incidence matrices relating observations to fixed and random effects, respectively; b is the vector of fixed effects, including the intercept and site effects; u is the vector of random effects; and e is the vector of residuals. The random effects included additive genetic effects, genotype-by-environment interaction effects, and experimental design factors such as replication, row, and column effects. For simplicity, only the additive genetic effects are shown explicitly in Equation (1).

The expectations of additive genetic effects, residuals, and observations were assumed to be E(u)=0, E(e)=0, and E(y)=Xb, respectively. The joint variance structure of additive genetic effects and residuals was


(2a) $$\begin{array}{c} Var\left[\begin{array}{c} u\\ e \end{array}\right]=\left[\begin{array}{cc} G & 0\\ 0 & R \end{array}\right]\; \end{array}$$

In the baseline model, the variance of additive genetic effects was specified as $G=A\sigma_{u}^{2}$ where A is the pedigree-based additive relationship matrix and $\sigma_{u}^{2}$ is the additive genetic variance.

Residuals were initially assumed to have variance $R=I\sigma_{e}^{2}$, where I is an identity matrix of appropriate dimension. These assumptions were relaxed by allowing heterogeneous residual variances across test sites using a direct sum of site-specific residual matrices:


(2b) $$\begin{array}{c} R=\;{⊕}_{i=1}^{t}R_{i}\; \end{array}$$

Where ⊕ denotes the direct sum operator, and $R_{i}$ is the residual covariance matrix for site *i* (Isik et al. 2017). Under these assumptions, the observation vector was assumed to follow a multivariate normal distribution: $y=MVN(Xb,\;ZAZ^{T}\sigma_{u}^{2}+R)$, where $Z^{T}$ is the transposed incidence matrix for additive genetic effects so that the variance of observations is $Var(y)=ZAZ^{T}\sigma_{u}^{2}+R$.

Henderson's (1982) mixed model equations were solved to estimate EBV:


(3) $$\begin{array}{c} \left[\begin{array}{c} \hat{b}\\ \hat{u} \end{array}\right]=\;\left[\begin{array}{c} X^{T}R^{-1}y\\ Z^{T}R^{-1}y \end{array}\right]\;{\left[\begin{array}{cc} X^{T}R^{-1}X & X^{T}R^{-1}Z\\ Z^{T}R^{-1}X & Z^{T}R^{-1}Z+A^{-1}\sigma_{u}^{-2} \end{array}\right]}^{-1} \end{array}$$

Here, $\hat{b}$ is the vector of best linear unbiased estimates of fixed effects, and $\hat{u}$ is the vector of best linear unbiased predictions of additive genetic effects, interpreted here as EBVs. Again, only the additive genetic effects are shown explicitly in Equation (3) for brevity.

#### Predictions of genomic breeding values using ssGBLUP

In this study, only a subset of the individuals was genotyped due to the cost of marker assays and logistical constraints (Table 1). To fully exploit all available phenotypic, pedigree, and genomic information, we applied single-step genomic BLUP (ssGBLUP), in a single linear mixed-model framework. The linear mixed model and assumptions were identical to those described in Equations (1) and (2), except that the additive genetic effects (u) were modeled using a hybrid relationship matrix (H), which integrates pedigree-based (A) and marker-based (G) genetic relationships matrices. In practice, the inverse of H is used because it is computationally more efficient to construct than H itself (Aguilar et al. 2010; Christensen and Lund 2010).


(4) $$\begin{array}{c} H^{-1}=A^{-1}+\left[\begin{array}{cc} 0 & 0\\ 0 & G^{-1}-A_{22}^{-1} \end{array}\right]\; \end{array}$$

where $A^{-1}$ is the inverse of the full A matrix, $A_{22}^{-1}$ is the inverse of the pedigree-based submatrix for genotyped individuals, and $G^{-1}$ is the inverse of the genomic relationship matrix for the same individuals. Additive genetic relationships based on pedigree are subtracted from marker-based relationships of genotyped individuals to prevent double counting.

#### Compatibility of genomic and pedigree-based relationships

The genomic relationships matrix (G**)** among genotyped individuals was estimated using the allele frequency method of VanRaden (2008). Let M be the matrix of gene content centered on 0, where each element takes the value −1, 0, or 1, corresponding to homozygous for the major allele, heterozygous, and homozygous for the minor allele, respectively. Marker genotypes were centered by subtracting twice the allele frequency at each locus ($2p_{i}$). The G matrix was computed as


(5) $$G=\;\frac{(M-2P){\left(M-2P\right)}^{T}}{2\sum p_{i}(1-p_{i})}\;$$

where $p_{i}$ is the minor allele frequency at marker *i*, P is a matrix of allele frequencies, $2\sum p_{i}(1-p_{i})$ represents the total marker-based additive genetic variance summed across all *m* loci.

For ssGBLUP, G must be compatible with the pedigree-based relationship matrix among genotyped individuals ($A_{22}$), which assumes a base population of founders having mean relationships of zero. To address incompatibility, first we aligned the G matrix with the $A_{22}$ matrix using the regression method according to Christensen et al. (2012);


(6) $$G_{w}=\alpha J+\beta G\;$$

where J is the unity matrix, *α* and *β* are the intercept and the slope, respectively. The regression parameters were estimated by equating the elements of 2 matrices, G and $A_{22}$ and the means of their diagonal elements as follows (Meyer et al. 2018);


(7a) $$\alpha =[\;\mathrm{tr}(A_{22})-\mathrm{tr}(G)]\;/n_{2}$$

(7b) $$\beta =\frac{[\mathrm{tr}(A_{22})-1^{\prime }A_{22}1]/n_{2}}{\;[\mathrm{tr}(G)-1^{\prime }G1]/n_{2}}\;$$

where tr(.) denotes the trace (sum of diagonal elements), $n_{2}$ is the number of genotyped individuals and 1′ is the transposed vector of unity. This scaling ensures that G and $A_{22}$ are on the same genetic base, enabling unbiased integration into the ssGBLUP model (Bermann et al. 2022). Then the G matrix was blended with the $A_{22}$ matrix to improve numerical stability and ensure invertibility. A scalar weighting parameter *λ* was applied in the $H^{-1}$ to scale the genomic and pedigree relationships to reduce the bias in predictions of GEBV (Aguilar et al. 2010).


(8) $$\begin{array}{c} H^{-1}=A^{-1}+\left[\begin{array}{cc} 0 & 0\\ 0 & \lambda G_{w}^{-1}-(1-\lambda )A_{22}^{-1} \end{array}\right]\; \end{array}$$

The parameter *λ* ranges from 0 and 1, where λ=1 assigns full weight on genomic relationships for genotyped trees, whereas smaller values of *λ* shrink genomic relationships toward the pedigree relationships, thereby increasing the contribution of $A_{22}$. Reducing *λ* can be advantageous for traits of lower heritability, or when SNP markers capture only a portion of the additive genetic variance (Meyer et al. 2018). We evaluated *λ* values of 0.9, 0.8, 0.7, 0.6, and 0.5 to assess the sensitivity of variance component estimates and the predictions of GEBV to the relative weighting of genomic information in $H^{-1}$. For stem volume, a value of *λ* = 0.50 was used in the final analysis, as equal weighting of marker- and pedigree-based relationships resulted in improved model performance. For stem straightness, the default value of *λ* = 0.90 was retained in the final analysis.

### GS validation

#### Scenario 1: ACE1 as training and ACE2 as validation

In this scenario, EBVs for the 2-generation ACE population were first estimated using the pedigree-based relationship matrix and phenotypes in the conventional ABLUP model. Next, the phenotypic records of the ACE2 population were masked and the ssGBLUP model was used to predict breeding values. The ssGBLUP model included phenotypes from ACE1 (2,076 genotyped trees) as a training set but not for the ACE2 (2,108 genotyped trees) as a validation set. The average genomic relationship between the training and validation sets was 0.036 (Supplementary Table 1).

#### Scenario 2: ACE1 and Mainline as training and ACE2 as validation

In this scenario, the objective was to understand the effect of a larger training set on the prediction accuracy. We first fit the ABLUP model using combined phenotypic data from the 2-generation ACE and Mainline populations to obtain EBVs. Next, the phenotypic records of the ACE2 population were masked and the ssGBLUP model was used to predict breeding values. This ssGBLUP model used ACE1 and Mainline as the training set (9,071 genotyped trees) and ACE2 population as the validation set (2018 genotyped trees). The average genomic relationship between the training and validation sets was 0.024 (Supplementary Table 1).

#### Scenario 3: ACE as training and Mainline population as validation

In this scenario, the 2-generation ACE population served as the training set, while the Mainline population was designated as the validation set. First, EBVs were estimated for all phenotyped trees as in Scenario 2. Next, phenotypic records for 6,140 genotyped trees in the Mainline population were masked. The ssGBLUP model was used to predict GEBVs using the genotyped Mainline trees as a validation set and the training population consisted of ACE trees. The average genomic relationship between the training and validation sets was 0.021 (Supplementary Table 1).

#### The impact of genetic relatedness on prediction

The degree of genetic relatedness between the training and validation sets is a critical factor influencing the accuracy of GS (Isik 2022; Lauer et al. 2022; Shalizi et al. 2022). This is because SNP markers essentially capture genetic relationships more effectively than pedigree-based relationships (Forni et al. 2011). To evaluate this effect, the Mainline population was partitioned into validation subsets according to their average genomic relationship with the ACE training set. Twelve subsets were created, with average genomic relationships of 0.011 to 0.036 to the ACE population. Model validation statistics using the ACE as the training and the Mainline as validation were calculated separately for each subset to evaluate the impact of relatedness between the training and validation sets.

#### Validation metrics

We used linear regression analysis to evaluate the accuracy and potential bias of GEBVs in 2 validation populations (Legarra and Reverter 2018). In this analysis, EBVs of the validation set were regressed on their corresponding GEBVs, and several key validation metrics were derived:

- *Prediction accuracy*: calculated as the Pearson correlation between EBV and GEBVs. The EBVs are default selection unit in conventional methods, so we used them as the baseline.
- *Regression slope*: reflects prediction bias defined as the difference between means of true and predicted breeding values $\bar{u}-\hat{u}$. This is the slope of regressing EBV on GEBV. A slope of 1 indicates unbiased prediction, values smaller than 1 suggest inflation (overestimation of GEBVs), while values greater than 1 suggest deflation (underestimation of GEBVs).
- *Root mean squared error* (*RMSE*): quantifies the squared average prediction difference between EBV and GEBV and calculated here as $\mathrm{RMSE}=\sqrt{\frac{1}{n}\overset{n}{\underset{i=1}{\sum }}\;{\left(\mathrm{EB}V_{i}-\mathrm{GEB}V_{i}\right)}^{2}}$.
- *Coefficient of determination* (*R^2^*): measures the proportion of variance in EBVs explained by GEBVs and obtained from linear regression.

## Results

### Genetic parameter estimates and response to selection

Genetic parameter estimates are reported from ABLUP and ssGBLUP models using a scaling factor (*λ*) of 0.50 for volume and 0.90 for straightness (Table 2). Genetic parameter estimates from the 2 models were similar and not statistically different. Individual-tree narrow-sense heritability for stem volume was low (0.11 to 0.13), whereas heritability for stem straightness was moderate (0.19 to 0.22). Additive genetic correlations between pairs of sites, reflecting the magnitude of genotype-by-environment interaction, were moderate for stem volume (0.70 for ABLUP and 0.66 and for ssGBLUP) and higher for stem straightness (0.83 and 0.87, respectively).

**Table 2.** Estimates of variance components, individual-tree narrow-sense heritability (*h*^2^), and additive genetic correlation among sites (GxE) for stem volume and stem straightness in a 2-generation Atlantic Coastal Elite (ACE) population of loblolly pine.

	Stem volume		Stem straightness	
Parameters	ABLUP	ssGBLUP	ABLUP	ssGBLUP
Additive variance	33 ± 2.7	30 ± 2.5	0.24 ± 0.015	0.29 ± 0.018
Additive × site (GxE)	15 ± 1.9	23 ± 2.7	0.03 ± 0.007	0.06 ± 0.011
Residual variance	216 ± 3.7	222 ± 3.6	0.96 ± 0.015	0.95 ± 0.015
Heritability (*h*^2^)	0.13 ± 0.009	0.11 ± 0.009	0.19 ± 0.010	0.22 ± 0.012
GxE correlation	0.70 ± 0.032	0.66 ± 0.034	0.87 ± 0.023	0.83 ± 0.028

Results are presented for pedigree-based BLUP (ABLUP) and single-step genomic BLUP (ssGBLUP) models. For ssGBLUP, marker- and pedigree-based relationship matrices were combined using scaling factor (*λ*) of 50% for stem volume and 90% for stem straightness. Estimates are followed by their standard errors (± SE).

**Fig. 2.**
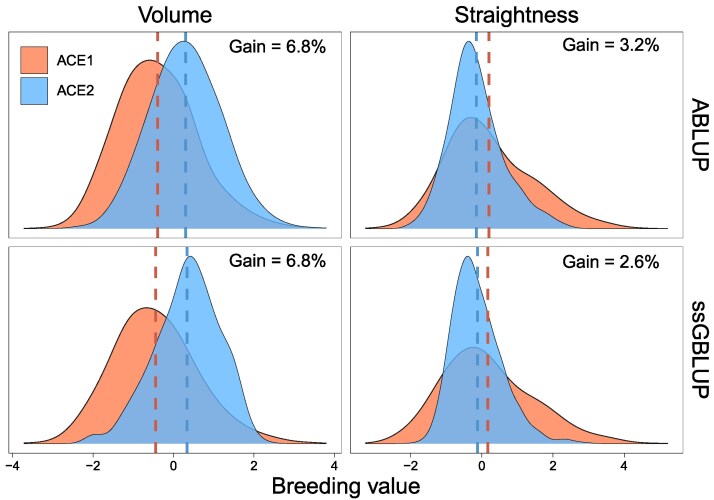
Distributions of breeding values across 2 generations of Atlantic Coastal Elite Population (ACE1 and ACE2) of loblolly pine, estimated using pedigree-based (ABLUP) and single-step genomic (ssGBLUP) models. Dashed vertical lines are population means. For stem volume, breeding values distributions shift rightward from ACE1 to ACE2 under both models, corresponding to a genetic gain of 6.8%. For stem straightness, improvement is indicated by a leftward shift in breeding value distributions, as smaller values correspond to straighter stems.

The distributions of breeding values (EBVs and GEBVs) for the first (ACE1) and second (ACE2) generations are shown in Fig. 2. Genetic gain estimates for stem volume were 6.8% per generation under both models, whereas gains for stem straightness were 3.2% for EBVs and 2.6% for GEBVs. Similar shifts in average EBV and GEBV across generations indicated that conventional selection and GS achieved comparable overall genetic gain across 2 ACE generations. The narrower and more peaked GEBV distributions observed in ACE2 population indicate greater shrinkage relative to EBVs, reflecting reduced variance in genomic predictions in the absence of phenotypes.

Assuming a breeding cycle length of 8 yr for GS and 12 yr for conventional selection, the annual rate of genetic gain was substantially higher using GS for stem volume and for stem straightness. This represents an approximately 20% to 50% increase in annual genetic gain attributable to cycle-time reduction.

### Validation of GS

**Fig. 3.**
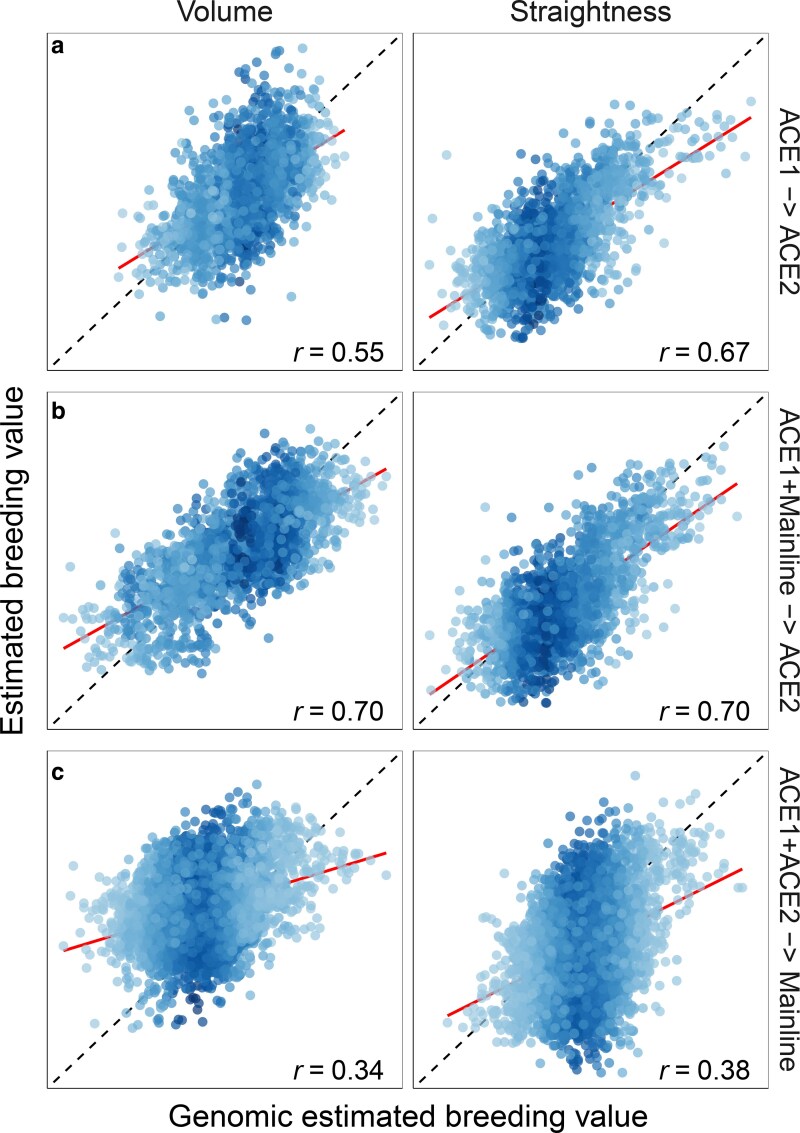
Relationships between genomic estimated breeding values and pedigree-based estimated breeding values for stem volume and stem straightness under 3 validation scenarios. The dashed black line indicates the 1:1 relationship, and the solid line shows the fitted regression. Correlation coefficients (*r*) represent prediction accuracy for each scenario. a) Scenario 1: ACE1 as training and ACE2 as validation set. The training and validation sets were genetically closely related to each other. b) Scenario 2: ACE1 + Mainline (9,071 genotyped trees) as training and ACE2 as validation set. A larger training population size increased the prediction accuracy. c) Scenario 3: ACE1 + ACE2 as training and Mainline population as validation set. Lower accuracy in this scenario reflects weak genetic relatedness between training and validation sets.

In the first validation scenario, the ACE1 served as the training population and ACE2 as the validation population. Prediction accuracy, measured as the correlation between EBVs and GEBVs, was moderate for stem volume (r=0.55) and high for stem straightness (r=0.67) (Fig. 3a). Regression slopes for volume ($b_{1}=0.64)$ and stem straightness ($b_{1}=0.66)$ indicated inflation of GEBVs (Table 3), implying that the ssGBLUP model slightly overestimated the genetic merit of individuals at the lower tails of the distribution and underestimated those at the upper tail. Although this suggests moderate bias in the scale of GEBVs, their ability to rank individuals remains strong.

**Table 3.** Validation statistics for genomic prediction of stem volume and stem straightness in loblolly pine using the second-generation Atlantic Coastal Elite (ACE2) and Coastal Mainline populations.

Training set	Validation set	Bias (slope)	*R* ^2^	RMSE
**Stem volume** (cubic decimeter)				
ACE1	ACE2	0.64	0.30	3.17
ACE1 + Mainline	ACE2	0.59	0.49	1.17
ACE1 + ACE2	Mainline	0.32	0.11	2.00
**Stem straightness** (1 to 6 scale)				
ACE1	ACE2	0.66	0.45	0.20
ACE1 + Mainline	ACE2	0.72	0.49	0.22
ACE1 + ACE2	Mainline	0.52	0.14	0.30

Model performance is reported for alternative training–validation combinations and summarized using the regression slope (bias), coefficient of determination ($R^{2}$), and root mean squared error (RMSE). For ssGBLUP, marker- and pedigree-based relationship matrices were combined using scaling factor (*λ*) of 50% for stem volume and 90% for stem straightness.

In the second scenario, ACE1 and the Mainline population were combined to form a larger training set, with ACE2 retained for validation. The increase in training population size substantially improved prediction accuracy for both traits (r=0.70) (Fig. 3b). The GEBV inflation persisted for stem volume ($b_{1}=\;0.59$) but reduced for straightness ($b_{1}=\;0.72$) (Table 3). For both traits, GEBV explained 49% of the variation in EBV $\left(R^{2}=0.49\right)$.

In the third scenario, the combined ACE population (ACE1 + ACE2) was used for training, and the Mainline population served as the validation set by masking phenotypes of all genotyped individuals. Prediction accuracy declined markedly for stem volume (r=0.34) and stem straightness (r=0.38), accompanied by further reductions in regression slopes, indicating increased inflation of GEBVs (Fig. 3c). This decline reflects the lower average genetic relatedness between ACE and Mainline families (Supplementary Table 2).

**Fig. 4.**
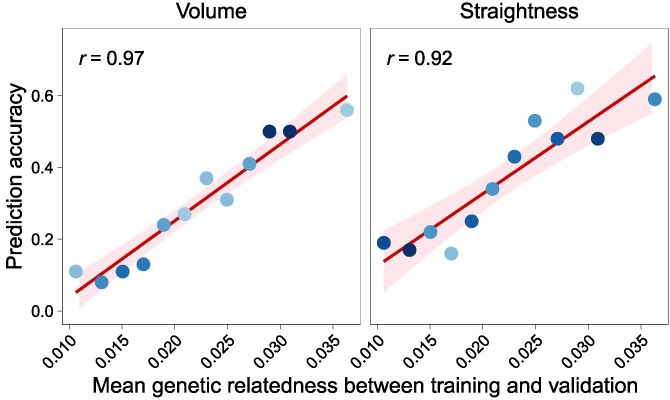
Relationship between mean genomic relatedness of training and validation populations and prediction accuracy of genomic estimated breeding values (GEBVs) for stem volume and stem straightness. Each point represents a validation subset from the Coastal Mainline population, with the Atlantic Coastal Elite (ACE) population used as the training set. Prediction accuracy increased with greater genetic relatedness between training and validation populations.

Prediction accuracy was strongly associated with mean genetic relatedness between training and validation sets when using the ACE population to predict GEBVs for the Mainline population (Fig. 4). The correlation between mean genetic relatedness and prediction accuracy was 0.97 for stem volume and 0.92 for stem straightness. This finding highlights the critical role of genetic connectivity for reliable genomic prediction.

### Scaling genetic relationships

Scaling the G and $A_{22}$ relationship matrices with the weight factor (*λ*) in the construction of $H^{-1}$ had consistent positive effects on genetic parameter estimates and genomic prediction performance in the ACE population. As *λ* decreased, thereby increasing the relative contribution of pedigree relationships, heritability estimates increased, prediction accuracy improved, and bias in GEBVs was reduced for both traits (Table 4).

**Table 4.** Effect of the scaling factor (*λ*) applied to marker- and pedigree-based genetic relationships in single-step genomic best linear unbiased prediction (ssGBLUP) on estimates of narrow-sense heritability and genomic prediction validation statistics for stem volume and stem straightness in the Atlantic Coastal Elite (ACE) population.

Lambda	Heritability	Prediction accuracy	Bias (slope)	*R* ^2^	RMSE
**Stem volume** (cubic decimeter)					
0.90	0.08 ± 0.007	0.51	0.54	0.26	3.55
0.80	0.09 ± 0.008	0.52	0.58	0.27	3.31
0.70	0.10 ± 0.008	0.53	0.60	0.28	3.19
0.60	0.11 ± 0.009	0.54	0.63	0.29	3.15
0.50	0.11 ± 0.009	0.55	0.64	0.30	3.17
**Stem straightness** (1 to 6 scale)					
0.90	0.22 ± 0.012	0.67	0.66	0.45	0.20
0.80	0.24 ± 0.013	0.69	0.70	0.48	0.20
0.70	0.25 ± 0.013	0.71	0.74	0.50	0.20
0.60	0.26 ± 0.013	0.72	0.78	0.52	0.17
0.50	0.26 ± 0.013	0.74	0.81	0.54	0.17

Results are shown for Scenario 1, with ACE1 used as the training population and ACE2 as the validation population. Validation statistics include prediction accuracy, regression slope (calibration), coefficient of determination ($R^{2}$), and root mean squared error (RMSE).

For stem volume, reducing *λ* (more weight on pedigree information) led to higher heritability and modest but consistent gains in prediction accuracy (0.51 to 0.55), accompanied by regression slopes closer to unity and improved overall model fit. Improvements tended to plateau at intermediate *λ* values, indicating diminishing returns from further increasing the weight on pedigree information. Collectively, these results support the use of λ=0.50 in the construction of $H^{-1}$ to improve estimation of GEBVs for stem volume in ssGBLUP model.

For stem straightness, the response to scaling was stronger. Lower *λ* values (more weight on pedigree) resulted in clear increases in prediction accuracy and substantial reductions in GEBV inflation, along with improved model fit. For example, heritability increased from 0.22 to 0.26, while the prediction accuracy increased from 0.67 to 0.74 when the lambda decreased from 0.90 to 0.50. Compared with stem volume, genetic parameter estimates for straightness were less sensitive to scaling, consistent with its higher heritability.

## Discussions

This study validates the practical utility of GS across 2 generations in loblolly pine, an economically important forest tree species in the southern United States. With breeding, recombination can erode linkage disequilibrium between markers and causal loci; however, the prediction accuracies observed here were comparable to those reported in early GS studies based on cross-validation within the same generation (Lauer et al. 2022; Shalizi et al. 2022).

Estimates of variance components and heritability were similar between ABLUP and ssGBLUP models, particularly after genomic relationships were scaled to be compatible with pedigree-based genetic relationships. Genetic gain across generations estimated from GS was comparable to that from conventional selection.

We identified 3 major factors affecting GS prediction accuracy: genetic relatedness between training and validation populations, training population size, and scaling of genomic relationship matrices. In livestock, GS models trained within one breed often perform poorly in unrelated breeds because predictive ability depends on tracking recent shared haplotypes (Lund et al. 2014; Wiggans et al. 2017). GS studies in forest trees anticipated the importance of relatedness using cross-validation approaches (Isik 2022; Lauer et al. 2022; Shalizi et al. 2022), but true across-generation validation studies are only now emerging (Simiqueli et al. 2023). In our study, increasing mean relatedness between training and validation sets resulted in a linear increase in prediction accuracy from approximately 0.10 to 0.60, with no indication of a plateau. These results highlight the importance of investing in training populations that are well connected to the target breeding population.

Increasing the training population size from approximately 3,000 to 9,000 genotyped trees substantially improved prediction accuracy for stem volume, a trait with modest heritability (*h*^2^ ≈ 0.10), and resulted in a smaller improvement for stem straightness, which had higher heritability (*h*^2^ ≈ 0.20). When genetically connected to the validation population, larger training populations provide more information to estimate additive genetic effects from realized relationships, particularly within large full-sib families where markers capture Mendelian sampling variance (Hayes et al. 2009). The optimal number of genotyped individuals for model training depends on population diversity and effective population size, both of which are typically large in forest tree breeding programs (Beaulieu et al. 2014; Ukrainetz and Mansfield 2020). Theoretical derivations and simulation studies have been presented to estimate the appropriate training population size for varying population characteristics (Meuwissen 2009; Lee et al. 2017), and Beaulieu et al. (2024) presented a meta-analysis of different tree breeding studies.

Single-step genomic best linear unbiased prediction represents the most practical framework for implementing GS in operational tree breeding programs. This approach is now standard in livestock breeding, where the theory underlying the scaling of genomic and pedigree relationships is well established (VanRaden 2008; Christensen and Lund 2010; Bermann et al. 2022). Compared with 2-stage approaches, ssGBLUP is simpler, accounts for selection, and can reduce bias in GEBVs, provided that relationship matrices are properly scaled (Vitezica et al. 2011; Legarra et al. 2014). Despite its importance, appropriate scaling of genomic relationships has received limited attention in forest trees. Our results show that scaling can have a substantial, trait-dependent impact on variance components and prediction accuracy.

We consistently observed inflation (regression coefficients < 1) when EBVs of genotyped individuals were regressed on their GEBVs, even after scaling genomic and pedigree relationship matrices and accounting for residual polygenic effects (Christensen and Lund 2010). Inflated predictions were observed in a hybrid eucalyptus population, although those analyses were limited to a single generation and smaller population sizes (Cappa et al. 2019). In dairy cattle, bias in GEBVs of test bulls was reduced when genotyped bulls without progeny records were excluded (Koivula et al. 2018). Inflation of GEBVs is generally attributed to incompatible scaling between genomic and pedigree relationships, overweighting of genomic information, incomplete capture of additive genetic variance by markers, and limited relatedness between training and validation populations (VanRaden 2008; Misztal et al. 2009; Christensen and Lund 2010; Vitezica et al. 2011). In contrast, deflation observed in US dairy cattle has been linked to incomplete pedigree information and strong selection pressure (Misztal and Legarra 2017).

GS offers several important benefits for forest tree breeding. In addition to shortening the breeding cycles by enabling earlier selection of superior individuals before long-term field phenotypes are fully expressed, it can also increase selection accuracy, especially for traits that are expensive, difficult, or take years to measure, such as sawtimber and wood quality. GS captures realized genetic relationships more effectively than pedigree alone, helps correct missing or incomplete pedigree information, and can improve selection within and across generations (Isik 2022; Walker et al. 2022). Without DNA markers, offspring from the same cross would have the same mid-parent breeding value unless they have their own phenotype or their progenies phenotyped. By distinguishing Mendelian sampling among full-sibs, GS provides an additional source of information for ranking individuals within families early. Together, these advantages can increase the rate of genetic gain per unit time and make breeding programs more efficient, provided that genotyping costs are reasonable and the data to build the prediction models are of high quality.

### Within-family prediction and selection

The context of GS described here is the selection of progeny to establish the next-generation breeding population, as opposed to identifying individuals for plantation deployment. Conifer tree breeding populations are typically composed of many crosses, each represented by relatively modest family sizes (Isik and McKeand 2019). The emphasis in these systems is on capturing additive genetic variation for multiple complex traits, rather than exploiting hybrid vigor or combining favorable traits from different species. In this respect, the breeding objective is more analogous to dairy cattle breeding than to many agronomic crop or eucalyptus breeding programs. After progeny testing, parents with highest breeding values are included in seed orchards, and seeds from those orchards are used to establish operational plantations (McKeand 2019).

In contrast, many domesticated plant breeding programs, such as maize, and rice, are designed to exploit heterosis, where recombinant inbred lines are crossed to generate large populations for selection (Windhausen et al. 2012). Similarly, eucalyptus breeding often relies on interspecific hybridization strategies, where many offspring from a limited number of crosses are cloned and field tested (Grattapaglia 2017). In such systems, the primary focus is deployment rather than recurrent population improvement. Comparable approaches also exist in some conifer tree breeding programs where vegetative propagation or somatic embryogenesis enables the mass production of selected genotypes from specific crosses for deployment (Lenz et al. 2020).

Although within-family prediction has been discussed in some forest trees (Papin et al. 2024), it has generally been a relatively small part of those investigations. For example, Ukrainetz and Mansfield (2020) evaluated within-family accuracy, but their results did not provide clear support for routine operational use. Beaulieu et al. (2014) also discussed this issue briefly, citing maize recombinant inbred line crosses, but did not directly evaluate within-family GS. Overall, these scenarios are more relevant to deployment than selecting individuals to form the next breeding population.

Lower within-family accuracies are commonly reported in the literature (Ukrainetz and Mansfield 2020; Walker et al. 2022, Papin et al. 2024). Inadequate family sizes in the training population can be a reason for poor accuracy within-families (Papin et al. 2024). It remains unclear whether these reflects a limited ability of markers to capture Mendelian sampling within families, or simply the imprecision of the phenotypic data used to estimate breeding values, which often rely on a single field observation. When the reference EBV itself is imprecise, a low within-family correlation between EBV and GEBV does not necessarily indicate poor performance of the genomic prediction model, although that may be a contributing factor. Another likely explanation is the inherently lower within-family heritability associated with within-family selection relative to mass selection, which reduces the expected prediction accuracy (Isik et al. 2003). This concern is less relevant when young offspring have not yet expressed the target traits and GS provides the best available basis for predicting their breeding values.

### Implications for loblolly pine breeding

This study provides the first evaluation of genomic prediction across generations in loblolly pine and highlights the importance of developing large, genetically connected training populations supported by reliable pedigrees and high-quality phenotypes. With a well-developed training population, recurrent GS can be effective in loblolly pine breeding. In the North Carolina State University Cooperative Tree Improvement Program, development of a GS training population began in 2019 with genotyping approximately 4,000 trees in the elite population described in this study, followed by targeted genotyping of the Mainline population (fourth-cycle progeny tests) to reach a total of approximately 11,000 genotyped trees. The Mainline population consisted of over 55,000 progeny test trees, and genotyping focused on families contributing fifth-cycle selections. This training population is expected to provide adequate prediction accuracy to recurrent GS.

The current GS strategy envisions genotyping approximately 4,000 full-sibling seedlings per year from about 100 controlled crosses beginning in 2026, with roughly 3% of young trees selected as parents for breeding. All genotyped seedlings will be established in replicated field trials to generate high-quality phenotypic data to retrain models for continued improvement of genomic predictions. Some programs that employ clonal forestry have suggested using GS to discard seedlings from clonal testing, effectively increasing the selection intensity or reducing the testing load (Grattapaglia 2022; Balocchi et al. 2023). In our program, we expect retaining genotyped individuals for later phenotyping as continued validation and model retraining to be a better investment.

The loblolly pine recurrent GS strategy implemented by the Cooperative Tree Improvement Program emphasizes open-pollinated progeny testing of individuals selected based on GEBVs to rank them for deployment. Before committing substantial resources to these selections, such as establishing hundreds of copies in seed orchards, their genetic merit should be validated through progeny testing rather than EBVs or GEBVs.

Under conventional breeding, a full selection cycle requires approximately 12 yr (Isik and McKeand 2019). In contrast, a recurrent GS strategy has the potential to reduce the breeding cycle by approximately 4 yr, increasing the annual rate of genetic gain up to 50%. However, several biological and logistical challenges remain for full implementation of GS. In conventional breeding, cuttings from selected trees are typically collected at age 6 yr and grafted into the crowns of mature trees in the orchard to induce strobili production for breeding. Under the RGS strategy, cuttings would be collected from seedlings at 1 or 2 yr of age. It remains unclear whether grafts from such young selections will produce strobili as timely as those from older trees. Consequently, the anticipated 4 or 5-yr reduction in breeding cycle length from GS assumes onset of flowering similar to conventional selections, an assumption that is currently being empirically tested through grafting and flower induction experiments.

## Conclusions

This study demonstrates that GS could effectively bypass progeny testing in loblolly pine breeding programs, providing a clear pathway to increased rates of genetic gain. Prediction accuracy was strongly influenced by training population size, genetic relatedness between training and validation populations, and the scaling of genomic and pedigree relationship matrices. These results emphasize the importance of well-developed training populations and consistent statistical frameworks. Building on these findings, plans are underway to implement GS routinely in loblolly pine breeding programs beginning in 2026.

## Supplementary Material

jkag122_Supplementary_Data

## Data Availability

Data statistics are provided in Table 1. Approximate locations of field test sites are provided in Supplementary Fig. 1. The heatmap of genetic relationships is provided in Supplementary Fig. 2. Phenotype, pedigree, and genotype data sets are available on *figshare* repository: https://doi.org/10.6084/m9.figshare.31126867. Supplemental material available at G3 online.
